# The Impact of ECT on mood symptoms: 50 patients were assessed using Hamilton Depression rating scale at the start, mid point and end of ECT. These results showed a clear reduction in depression symptoms as ECT progressed.

**DOI:** 10.1192/j.eurpsy.2023.1938

**Published:** 2023-07-19

**Authors:** D. Collins

**Affiliations:** Yare Ward, Hellesdon Hospital, Drayton High Road, NSFT/NHS, Norwich, United Kingdom

## Abstract

**Introduction:**

ECT is a recognised, safe and effective treatment for severe/psychotic depression. Neuro and functional brain imaging has indicated what specific changes take place with ECT and at what stage. This study aims to explore the specific impact on depression and mood symptoms as ECT progresses, using longer version of Hamilton Depression Rating Scale at star, mid poitn and end point of ECT.

Patient themselves are often keen advocates of ECT (if they found it effective and tolerable).

**Objectives:**

specifically to consider the impact of ECT on mood and depressive symptoms as ECT progresses.

This is to reconsider and reflect on if ECT is effective and also to consider at what satge of treatment ECT appears to have the greatest impact or brings about the greatest change

All patients having ECT will be assessed and clerked as per routine but will also be assessed using Hamilton Depression Rating Scale (17 item version), an observer rated scale to assess severity of depressive symptoms. This will be used with all ECT patients, regardless of diagnosis or indication for ECT. Basic demographic details, diagnosis, co morbidities and previous ECT recorded.

Findings

**Methods:**

Patients are are ideally assessed before ECT has started to give a baseline/true reflection of their depressive illness and then at the mid point (around session 6) and then at the end of treatment (ideally session 12).

Hamilton depression Rating scale (HDRS) longer version is used - 17 questions with scores out of 40. Higher scores indicate more severe depression. HDRS is an observer rated scale, with numerous questiosn about biological symptoms of depression and is a well established assesment tool. The same rater was used to try to rule out observer bias.

**Results:**

Patients had a significant drop in HDRS scores as ECT progressed, with the biggest drop being between the start of ECT and the midpoint. Thsi trend continued from mid to end point but with a less steep gradient.

mean HDRS scores at start were 24/40 (indicting severe depression).

Mean HDRS at midpoint 12/40 (indicating mild depression)

Mean HDRS at end opf ECT 5/40 (indicating nromal range/no depression)

**Conclusions:**

ECT works for mood symptoms associated with depression. All patients having ECT had a reduction in their HDRS scale as ECT progressed, most marked in the 1^st^ half but this trend continued in 2^nd^ half (but at slower rate). This was the case even for patients who were not having ECT for mood symptoms (eg for aggression or psychosis).

**Disclosure of Interest:**

None Declared

